# Liposomal encapsulation of cholecalciferol mitigates *in vivo* toxicity and delays tumor growth

**DOI:** 10.3389/fimmu.2025.1529007

**Published:** 2025-01-27

**Authors:** Miriam Ezcurra-Hualde, Sara Zalba, Ángela Bella, Leire Arrizabalaga, Aline Risson, Román García-Fuentes, Celia Gomar, Nuria Ardaiz, Virginia Belsue, David Ruiz-Guillamon, Alejandro Serrano-Alcaide, Ainara Salgado, Fernando Aranda, Maria J. Garrido, Pedro Berraondo

**Affiliations:** ^1^ Program of Immunology and Immunotherapy, Cima Universidad de Navarra, Cancer Center Clínica Universidad de Navarra (CCUN), Pamplona, Spain; ^2^ Navarra Institute for Health Research (IDISNA), Pamplona, Spain; ^3^ Department of Pharmaceutical Sciences, School of Pharmacy & Nutrition, University of Navarra, Pamplona, Spain; ^4^ Spanish Center for Biomedical Research Network in Oncology (CIBERONC), Madrid, Spain

**Keywords:** liposomal encapsulation, vitamin D_3_, anticancer efficacy, gene expression, tumor growth

## Abstract

**Introduction:**

Vitamin D_3_ (cholecalciferol) has demonstrated potential anticancer properties, but its clinical application is limited by associated toxicity at effective doses. This study investigated the use of liposomal encapsulation to increase the therapeutic efficacy of vitamin D_3_ while mitigating its toxicity.

**Methods:**

Liposomal vitamin D_3_ (VD-LP) was prepared via the film-hydration method and characterized for particle size, polydispersity index, encapsulation efficiency, and long-term stability. *In vitro* gene expression modulation was evaluated in monocytic THP-1 cells, and antiproliferative effects were assessed in HT29 (colorectal), BT474 (breast), and TRAMP-C1 (prostate) cancer cell lines. *In vivo* antitumor efficacy and toxicity were tested in a mouse model with subcutaneously implanted MC38 tumors. Tumor growth, survival rates, and serum calcium and phosphate levels were analyzed.

**Results:**

VD-LP demonstrated high encapsulation efficiency and stability over 90 days, with a consistent particle size of approximately 83 nm. VD-LP modulated immune-related and metabolic gene expression in THP-1 cells, including upregulation of antimicrobial peptides and vitamin D receptor genes. VD-LP showed superior antiproliferative effects compared to free vitamin D_3_ in all tested cancer cell lines. *In vivo*, VD-LP delayed tumor growth and improved survival without causing hypercalcemia, highlighting its favorable toxicity profile.

**Discussion:**

Liposomal encapsulation of vitamin D_3_ significantly improves its anticancer efficacy while mitigating toxicity, making it a promising strategy for future cancer therapies. VD-LP shows potential for enhanced therapeutic applications with reduced adverse effects, warranting further clinical exploration.

## 1 Introduction

Vitamin D, particularly its active form, vitamin D_3_ (cholecalciferol), is traditionally known for its role in calcium homeostasis and bone health (1). However, emerging evidence over the past few decades has revealed a much broader spectrum of biological functions, including modulation of the immune system, regulation of cell proliferation and differentiation, and a potential role in the prevention and treatment of various malignancies (2). Vitamin D_3_ exerts its effects primarily through the vitamin D receptor (VDR), a nuclear receptor that regulates the expression of numerous genes involved in cellular growth, immune responses, and metabolic processes (3).

Several epidemiological studies have suggested an inverse relationship between vitamin D levels and the incidence of various cancers, including colorectal, breast, and prostate cancers (2). *In vitro* and *in vivo* studies have further supported these observations, demonstrating that vitamin D_3_ can induce cell cycle arrest, promote apoptosis, inhibit angiogenesis, and modulate the tumor microenvironment. These anticancer effects are believed to be mediated through the activation of the VDR and subsequent transcriptional regulation of target genes involved in these processes (4).

Despite the promising anticancer properties of vitamin D_3_, its clinical application has been significantly hindered by its narrow therapeutic window (5). At the therapeutic doses required to exert anticancer effects, vitamin D_3_ can induce hypercalcemia, hypercalciuria, and other toxic effects, limiting its safe administration (6). Hypercalcemia resulting from vitamin D_3_ toxicity can manifest as nephrocalcinosis, renal failure, cardiac arrhythmias, and soft tissue calcification, posing significant risks to patients (7). The primary mechanism of this toxicity involves the dysregulation of calcium absorption and mobilization due to the overactivation of the VDR (8). Consequently, vitamin D_3_’s therapeutic potential is often overshadowed by its narrow therapeutic index, which limits its safe and effective use, especially in high doses required for treating conditions such as cancer. This challenge has prompted the exploration of various strategies aimed at enhancing its efficacy while minimizing toxicity.

One widely explored approach involves the development of vitamin D_3_ analogues with modified structures to retain biological activity while reducing the risk of hypercalcemia. These analogues, such as calcipotriol and paricalcitol, have shown promise in specific applications, particularly in dermatological and renal contexts. However, their broader use remains limited due to variable efficacy and potential off-target effects (9–13).

Another strategy involves combining vitamin D_3_ with agents that either enhance its antitumor activity or mitigate toxicity. For instance, pairing vitamin D_3_ with chemotherapeutic drugs or immune modulators has demonstrated synergistic effects in preclinical models. Despite these advances, such combinations require careful dose calibration to prevent adverse interactions and maintain safety (14–17).

Liposomal encapsulation of vitamin D_3_ represents a complementary and innovative approach that directly addresses the dual challenges of bioavailability and toxicity. Unlike free vitamin D_3_ or its analogues, liposomal formulations provide enhanced stability, targeted delivery, and sustained release, which collectively reduce systemic toxicity while improving therapeutic efficacy. While not the only solution, liposomal encapsulation offers unique advantages that make it a particularly promising strategy for clinical applications, especially in oncology. Liposomal encapsulation has emerged as a promising approach to improve the therapeutic index of various drugs, including those with poor solubility, poor stability, or significant toxicity. Liposomes are spherical vesicles composed of phospholipid bilayers that can encapsulate hydrophobic or hydrophilic drugs within their core or membrane. This encapsulation can protect the drug from degradation, increase its bioavailability, and provide controlled release, thereby reducing the frequency and dose of administration required (18).

The use of liposomal formulations has been particularly advantageous in cancer therapy, as they can facilitate targeted delivery to tumor tissues while minimizing systemic exposure and toxicity. Liposomes can preferentially accumulate in tumor tissues through the enhanced permeability and retention (EPR) effect, a phenomenon resulting from the leaky vasculature and poor lymphatic drainage typically associated with tumors. This targeted delivery not only enhances the therapeutic efficacy of the drug but also reduces the adverse effects on healthy tissues (19).

Given the challenges associated with the systemic administration of vitamin D_3_, liposomal encapsulation represents a potential strategy to enhance its anticancer effects while minimizing toxicity. Previous studies have demonstrated that liposomal vitamin D_3_ (VD-LP) can increase bioavailability, leading to more pronounced biological effects at lower doses (20). Furthermore, liposomal encapsulation may protect vitamin D_3_ from rapid degradation in the bloodstream, thereby extending its half-life and improving its therapeutic efficacy (21).

In this context, our study aimed to explore the potential of liposomal encapsulation to overcome the limitations associated with vitamin D_3_ therapy in cancer. We hypothesized that compared with VD, VD-LP would demonstrate enhanced stability, reduced toxicity, and improved anticancer efficacy. To test this hypothesis, we conducted a series of *in vitro* and *in vivo* experiments to evaluate the physicochemical properties, biological activity, and therapeutic potential of VD-LP. Our findings suggest that liposomal encapsulation not only enhances the delivery and efficacy of vitamin D_3_ but also significantly reduces the risk of hypercalcemia and other toxic effects, thus offering a safer alternative for clinical use.

## 2 Materials and methods

### 2.1 Vitamin D_3_ liposome preparation

Vitamin D_3_ (cholecalciferol) was purchased from Sigma (Spain); phosphatidylcholine-hydrogenated (HSPC), cholesterol (CH), and DSPE-PEG2K were purchased from Avanti Polar Lipids (USA). Other reagents were of analytical grade.

VD-LP was prepared via the film-hydration method. The methodology was based on a previous protocol (22, 23). Briefly, lipids and VD (HSPC: CH : VD: DSPE-PEG2K 60:35:4.5:5, molar ratio) were dissolved in a solution of chloroform:methanol [9:1 (v/v)]. The mixture was dried by rotary evaporation at 40°C (Büchi, Switzerland) to form a film, which was hydrated with HEPES buffer (pH 6.7) (Gibco, Waltham, Massachusetts, USA). Finally, for homogenization of the particle size, the liposomal solution was extruded through several polycarbonate membranes (from 200 nm in size to 80 nm in size). This protocol was also followed to formulate empty liposomes (HSPC: CH : DSPE-PEG2K, 65:35:5 molar ratio). The purification of the VD-LP was carried out by size exclusion chromatography using a PD10 column (loaded with Sephadex-25) (GE Healthcare, Madrid, Spain).

### 2.2 Characterization of vitamin D_3_ liposomes

The particle size, polydispersity index (PDI) and zeta potential were analyzed via laser diffractometry (DLS) using a Zetasizer Nano Series system (Malvern Instruments, UK). The lipid concentration was measured via a phosphate assay, and the encapsulation efficiency of VD was measured via a Nanodrop at 265 nm. The long-term stability of the VD-LP preserved at 4°C was assayed for 90 days.

For the morphological characterization of the formulations, transmission electronic microscopy (FE-SEM Zeiss Sigma 300 VP) was used to analyze the samples. Briefly, the samples suspended in ddH2O were laid on copper grids with a film of formvar (EMS, FF200-cu) for 2.5 min at room temperature. These samples were washed twice with ddH_2_O, and negative staining with 1% uranyl acetate for 15 s was performed.

### 2.3 Cell lines and culture

Tumor cell lines were grown with appropriate culture media. Adherent murine MC38 (University of Washington, Seattle, USA) and human HT29 (ATCC; HTB-38) colorectal cancer cells were cultured with RPMI 1640 (Gibco) supplemented with 10% (v/v) fetal bovine serum (FBS; Gibco), 100 IU penicillin and 100 µg/mL streptomycin (1% P/S; Gibco). The human BT474 breast cancer cell line (Hospital del Mar, Barcelona, Spain) was seeded with DMEM F12, 10% (v/v) FBS and 1% P/S. The murine TRAMP-C1 prostate cancer cell line (ATCC; CRL-2730) was cultured with DMEM F12, 10% (v/v) FBS, 1% P/S, 0.005 mg/mL bovine insulin (Sigma) and 10 nmol·L^-1^ dehydroisoandrosterone 90% (ACROS Organics, Thermo Fisher Scientific, Spain). The human monocytic leukemia cell line THP-1 (ATCC, TIB-202) was grown in suspension with RPMI 1640, 10% (v/v) FBS, 1% P/S and 0.9 µL/mL 2-mercaptoethanol. All the samples were incubated in a 5% CO_2_ humidified atmosphere at 37°C.

### 2.4 Evaluation of the modulation of gene expression

In a 96-well plate, 3×10^5^ cells/well/150 µL were seeded, and the cells were subsequently stimulated with 10 µL of VD-LP containing 0.25 µM vitamin D_3_, 10 µL of empty-liposomes (Empty-LP) or 0.25 µM VD mixed in a volume of 50 µL/well in triplicate. Vitamin D_3_ was prepared as a concentrated stock solution by dissolving it in absolute ethanol. The stock solution was diluted in the cell culture medium to achieve the final working concentration. The final ethanol concentration in the culture medium was less than 0.1%, a level that does not induce toxicity in the cells. This preparation ensured the solubility of VD_3_ and its bioavailability for comparison with the liposomal-encapsulated vitamin D_3_ formulation. For 24 h, the plates were incubated in a 5% CO_2_ atmosphere at 37°C.

RNA extraction was carried out with a Maxwell^®^ RSC simple RNA Tissue Kit and Instrument (Promega, Madison, Wisconsin, USA) following the manufacturer’s instructions. The RNA was quantified with a NanoDrop spectrophotometer (Thermo Scientific Wilmington, Delaware, USA).

Reverse transcription was performed from 300 ng of RNA with reverse transcriptase (Promega). Amplification from generated cDNA was performed with iQ SYBR Green Supermix (Bio-Rad, Hercules, California, USA). Specific forward (Fw) and reverse (Rv) primer sequences for each gene were purchased from Invitrogen (Thermo Fisher). Housekeeping gene RPLP0 Fw 5′-aacatctcccccttctcctt-3′ Rv5′-gaaggccttgaccttttcag-3′; cathelicidin antimicrobial peptide (hCAMP) Fw 5′-tgggcctggtgatgcct-3′ Rv 5′-cgaaggacagcttccttgtagc-3′. Vitamin D receptor (hVDR) Fw 5’-gtggacatcggcatgatgaag-3’ Rv 5’-ggtcg aggtcttatggtggg-3’. ATP-binding cassette subfamily D member 2 (ABCD2) Fw 5’-aatggaccagatcgagtgctg-3’ Rv 5’-tgggatagagggttttcagagc-3’. Fructose-1,6-biphosphatase 1 (FBP1) Fw 5’-cgcgcacctctatggcatt-3’ Rv 5’-ttcttctgacacgagaacacac-3’. Neuronal growth regulator 1 (NEGR1) Fw 5’-gcttgttgctcgaaccagtg-3’ Rv 5’-ccccttttctgaccatcatgtt-3’. The resulting amount of each transcript was expressed via the formula 2^ΔCt^.

### 2.5 RNA sequencing

RNA sequencing (RNAseq) analysis was performed to analyze MC38 tumors treated *in vivo* and THP-1 cells treated *in vitro*. MC38 tumors were isolated 48 h after the last dose. A total of 3.5 × 10^5^ THP-1 cells were stimulated with 10 µL of VD-LP containing 0.25 µM vitamin D_3_, 10 µL of Empty-LP or 0.25 µM VD in triplicate for 24 hours. In both cases, RNA was isolated with a Maxwell^®^ RSC simple RNA Tissue Kit and Instrument (Promega) and quantified with a NanoDrop spectrophotometer (ThermoScientific). Starting from the isolated RNA of the induction assay. The 20 ng/µL samples were sequenced by the Genomic Unit of the Center for Applied Medical Research (CIMA, University of Navarra). All the RNA samples were high-quality, with RIN values greater than 7. Library preparation was performed via the Illumina Stranded mRNA Prep Ligation Kit (Illumina) following the manufacturer’s protocol. All sequencing libraries were constructed from 100 ng of total RNA according to the manufacturer’s instructions. The protocol selects and purifies poly(A)-containing RNA molecules via magnetic beads coated with poly(T) oligos. Poly(A)-RNAs are fragmented and reverse transcribed into the first cDNA strand via random primers. The second cDNA strand is synthesized in the presence of dUTP to ensure strand specificity. The resulting cDNA fragments were purified with AMPure XP beads (Beckman Coulter), adenylated at 3′ ends and then ligated with uniquely indexed sequencing adapters. Ligated fragments are purified and PCR amplified to obtain the final libraries. The quality and quantity of the libraries were verified via a Qubit dsDNA HS Assay Kit (Thermo Fisher Scientific) and 4200 TapeStation with High Sensitivity D1000 ScreenTape (Agilent Technologies). Libraries were then sequenced via a NextSeq2000 sequencer (Illumina). Forty million pair-end reads (100 bp; Rd1:51; Rd2:51) were sequenced for each sample and demultiplexed via Cutadapt. RNA-seq was carried out at the Genomics Unit of CIMA.

### 2.6 *In vitro* proliferation assay

Analyses were carried out on a Real-Time Cell Analyzer xCELLigence. The HT29, BT474 and TRAMP-C1 cell lines were seeded in a 16-well E-Plate 16 PET (Aligent, Adelaida, South Australia, AUS) at a concentration of 3.5 × 10^4^ cells/well/100 µL. For 4–6 hours, readings were collected from the plate until exponential cell growth occurred. The treatments were subsequently added in duplicate: 0.25 μM VD, 10 μL of VD-LP containing 0.25 μM vitamin D_3_ and 10 μL of Empty-LP. Readings were collected for an additional 72–96 hours.

### 2.7 *In vivo* antitumor efficacy

C57BL/6J mice (female, 5 weeks old) were purchased from Harlan Laboratories (Barcelona, Spain) and maintained under a 12 h light/dark cycle with free access to food and water. After trypsinization of the MC38 cells and counting of the cells (98% viability) and previous shaving, each mouse received a subcutaneous injection in the flank of the right hind leg of 5 × 10^5^ cells resuspended in 100 µL of PBS and randomly assigned to treatment cohorts: control/HEPES, VD, VD-LP and Empty-LP. A dose of 30 µg of vitamin D_3_ was given to each mouse in the VD and VD-LP groups, and the equivalent quantity of lipids in Empty-LP and VD-LP was determined. All the treatments were administered intravenously through the lateral tail vein in a volume of 200 µL every 2 days for a total of 3 doses/mouse. The tumors were measured 2 days a week. Twenty-four hours after the last administration, serum samples were drawn to analyze the calcium (Ca^2+^) and phosphate (PO_4_^3-^) levels via a chemical analyzer (Cobas c311, Roche).

### 2.8 Statistical analysis

The software used for statistical analysis was GraphPad Prism version 8.0.2 (GraphPad Software, San Diego, California, USA). The data were analyzed via one-way ANOVA followed by ordinary ANOVA, the Kruskal-Wallis test, Sidak’s multiple comparisons test or the log-rank Mantel-Cox test. Significant differences *p ≤ 0.05, **p ≤ 0.01, ***p ≤ 0.001, ****p ≤ 0.0001.

## 3 Results

### 3.1 Characterization and stability of the VD-LP formulation

**Figure 1:**
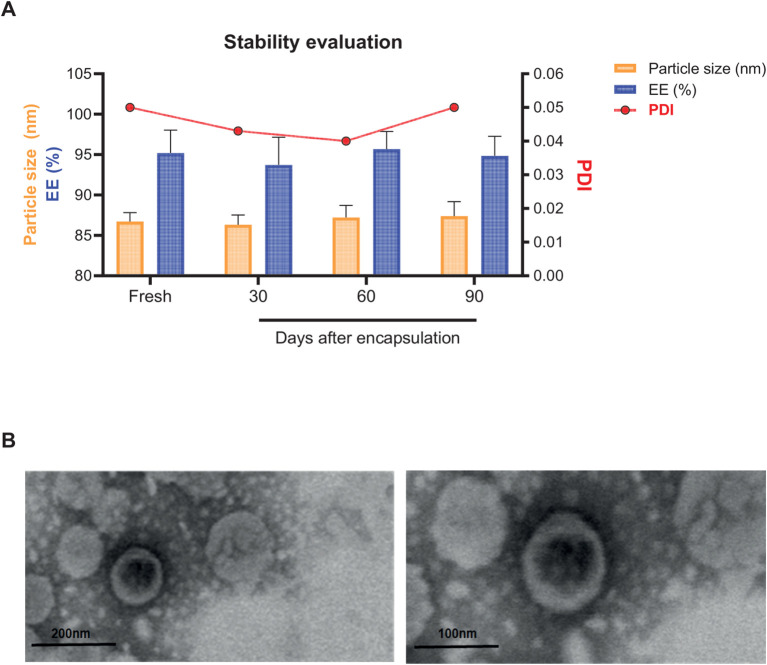
Characterization and stability of the VD-LP formulation. **(A)** Stability evaluation of VD-LP over 90 days at 4°C. The particle size (nm), polydispersity index (PDI), and encapsulation efficiency (EE%) were monitored and remained stable throughout the study period. The data represent the means ± SDs of three independent batches. **(B)** Transmission electron microscopy (TEM) images of the VD-LP showing spherical liposomes with sizes of less than 100 nm. The images confirmed the uniform morphology of the liposomes.

The physicochemical properties of the VD-LP formulation were evaluated over a 90-day period at 4°C to assess its stability. The particle size, polydispersity index (PDI), and encapsulation efficiency (EE) of VD-LP remained stable throughout the study. The mean particle size of the VD-LP was consistently less than 100 nm (mean ± SD: 82.69 ± 0.984 nm), with a PDI of 0.041 ± 0.012, indicating a narrow size distribution. The encapsulation efficiency was high, with a mean value of 94.85% ± 4.82% (**Figure 1A**, **Table 1**). Transmission electron microscopy (TEM) confirmed the spherical morphology of the liposomes and their uniform size distribution (**Figure 1B**). These results indicate that the VD-LP formulation is stable and uniform throughout the storage period.

**Table 1:** Physicochemical characterization of empty and VD liposomal formulations.

	Empty-LP	VD-LP
**Particle size (nm)**	96.72 ± 0.949	82.69 ± 0.984
**PDI**	0.046 ± 0.009	0.041 ± 0.012
**Zeta potential (mV)**	-6.24 ± 1.05	-24.6 ± 2.54
**EE (%)**	–	94.85 ± 4.82

PDI, polydispersity index; EE, encapsulation efficiency.

The data represent the means ± SDs of three independent batches.

### 3.2 Modulation of gene expression by VD-LP in THP-1 cells

**Figure 2:**
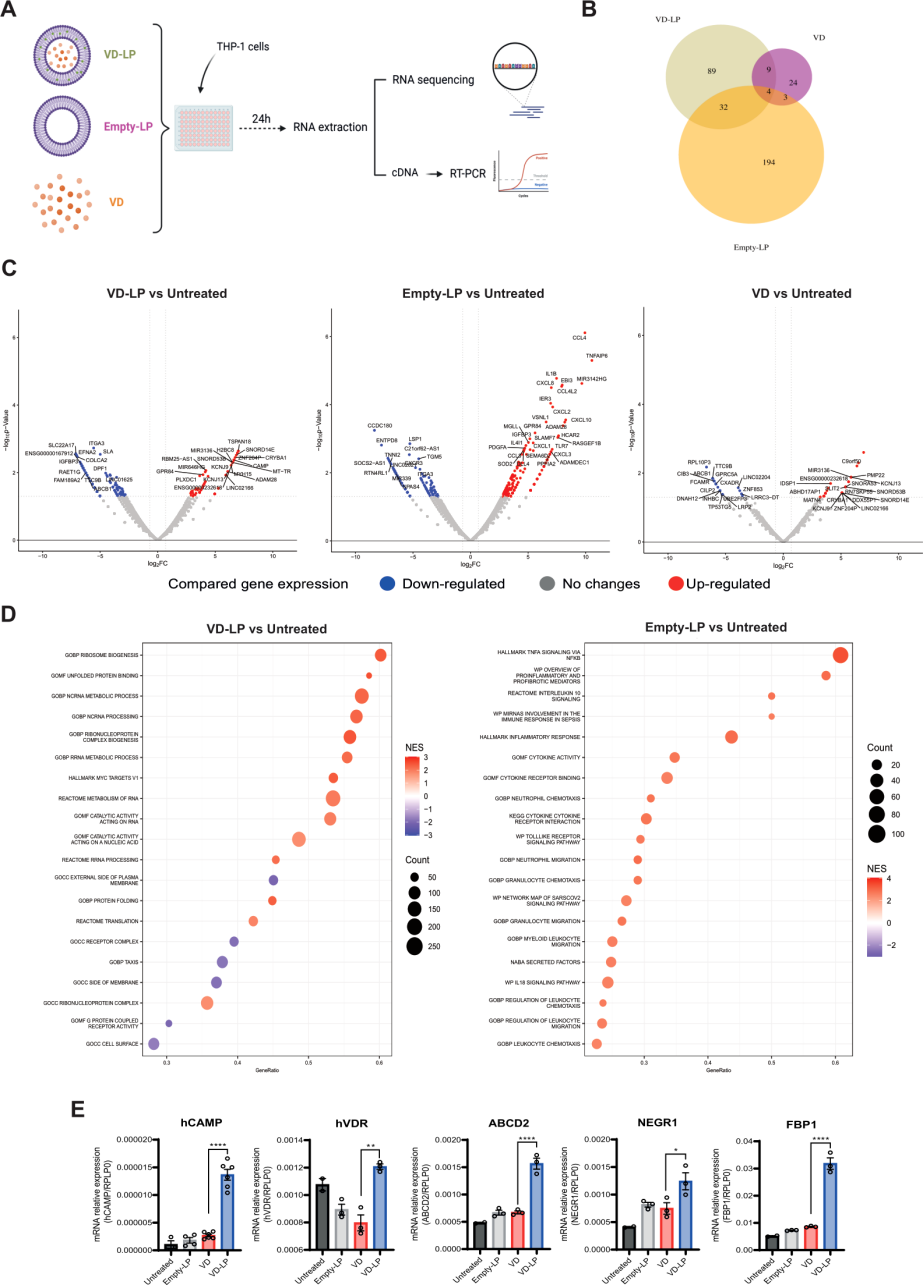
Modulation of gene expression by VD-LP in THP-1 cells. **(A)** A total of 300000 THP-1 cells were seeded in a 96-well plate and stimulated for 24 h with VD-LP, VD or Empty-LP. RNA was extracted for RNA-seq, and cDNA was generated for PCR. **(B)** Venn diagram representing the number of modulated genes in each experimental group. **(C)** Volcano plots representing down- and upregulated genes. **(D)** GSEA of differentially regulated pathways. **(E)** Real-time PCR analysis of cathelicidin antimicrobial peptide (hCAMP), vitamin D receptor (hVDR), ATP-binding cassette subfamily D member 2 (ABCD2), fructose-1,6-biphosphatase 1 (FBP1), and neuronal growth regulator 1 (NEGR1). One-way ANOVA with Sidak’s multiple comparisons test was used. *p ≤ 0.05, **p ≤ 0.01, ****p ≤0.0001.

To evaluate the biological activity of VD-LP, we performed gene expression analysis on human monocytic THP-1 cells following treatment with VD-LP, VD, or Empty-LP (**Figure 2A**). RNA-seq revealed significant differences in gene expression profiles among the treatment groups. Cells treated with free VD presented the fewest changes in gene expression, likely reflecting the limited ability of free VD to penetrate the cytoplasm and activate the vitamin D receptor (**Figures 2B, C**). In contrast, both VD-LP and Empty-LP had more pronounced effects on gene expression (**Figures 2B, C**). Notably, Empty-LP induced the greatest number of gene expression changes (**Figure 2B**), likely due to its promotion of inflammatory pathways, with TNF signaling being the most prominently upregulated pathway (**Figure 2D**). For VD-LP, the upregulated pathways were related primarily to metabolic processes. These findings indicate that incorporating vitamin D into liposomes helps prevent the activation of inflammatory responses while facilitating intracellular vitamin D_3_ activity (**Figure 2D**). To validate these findings, real-time PCR analysis was conducted, confirming that VD-LP treatment led to the upregulation of key genes such as cathelicidin antimicrobial peptide (hCAMP), vitamin D receptor (hVDR), ATP-binding cassette subfamily D member 2 (ABCD2), neuronal growth regulator 1 (NEGR1) and fructose-1,6-bisphosphatase 1 (FBP1). Specifically, fold-change increases in expression were: hCAMP 5-fold, hVDR 1.5-fold, ABCD2 2.3-fold, NEGR1 1.6-fold, and FBP1 3.7-fold (**Figure 2E**). Overall, VD-LP treatment effectively modulated the expression of genes involved in the immune response and metabolism in THP-1 cells.

### 3.3 Antiproliferative effects of VD-LP in cancer cell lines

**Figure 3:**
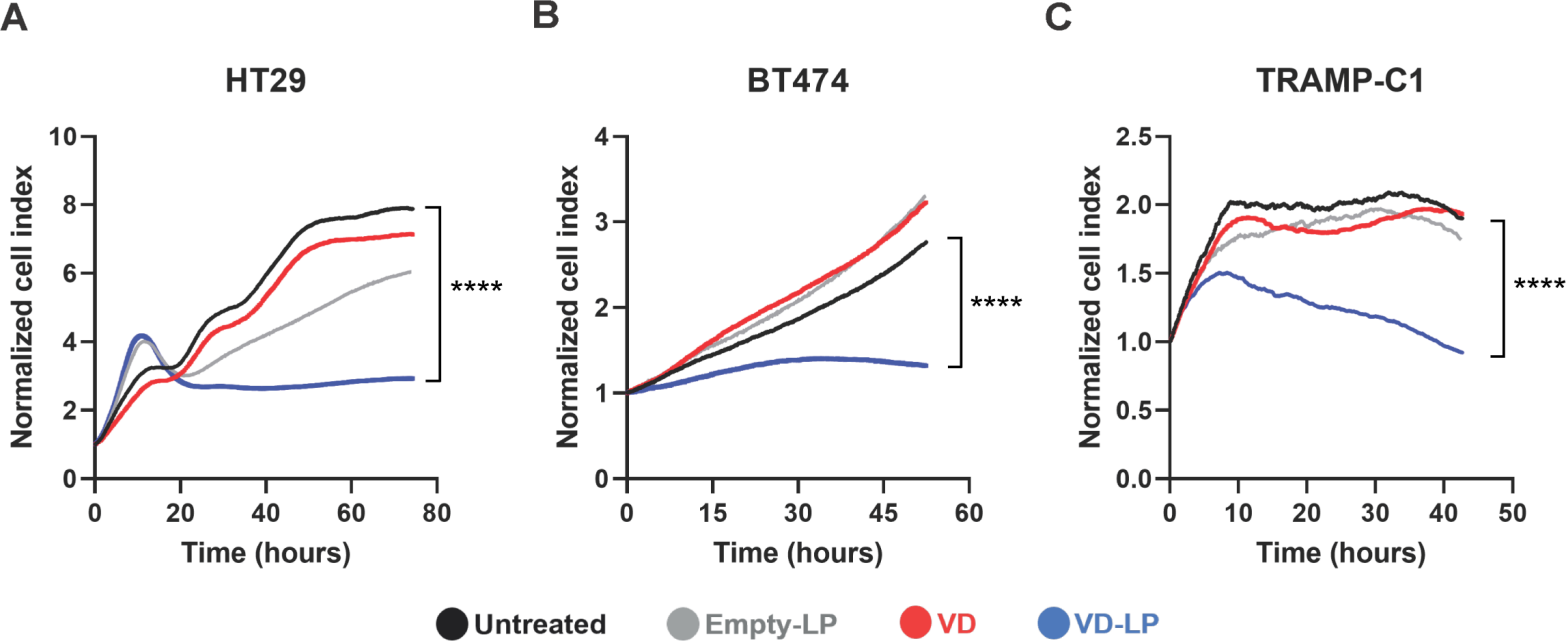
Antiproliferative effects of VD-LP in cancer cell lines. **(A–C)** Evaluation of the antiproliferative effects of VD-LP on the HT29 (human colorectal cancer), BT474 (human breast cancer), and TRAMP-C1 (prostate cancer) cell lines. The cells were treated with VD-LP, free VD, or Empty-LP or left untreated, and cell proliferation was monitored via xCELLigence real-time cell analysis. One-way ANOVA with the Kruskal-Wallis test; ****p ≤ 0.0001.

The antiproliferative effects of VD-LP were assessed in three cancer cell lines: HT29 (human colorectal cancer), BT474 (human breast cancer), and TRAMP-C1 (mouse prostate cancer). Using real-time cell analysis via the xCELLigence system, we found that compared with no treatment, VD-LP treatment significantly inhibited the proliferation of all three cell lines. At 40 hours, VD-LP treatment resulted in a significant reduction in cell proliferation: 86.4% in HT29 cells, 56.1% in BT474 cells, and 84.5% in TRAMP-C1 cells. These results demonstrate a pronounced sensitivity of HT29 and TRAMP-C1 cells to VD-LP, with a more moderate effect observed in BT474 cells (**Figure 3**).

### 3.4 *In vivo* antitumor efficacy and toxicity of VD-LP

**Figure 4:**
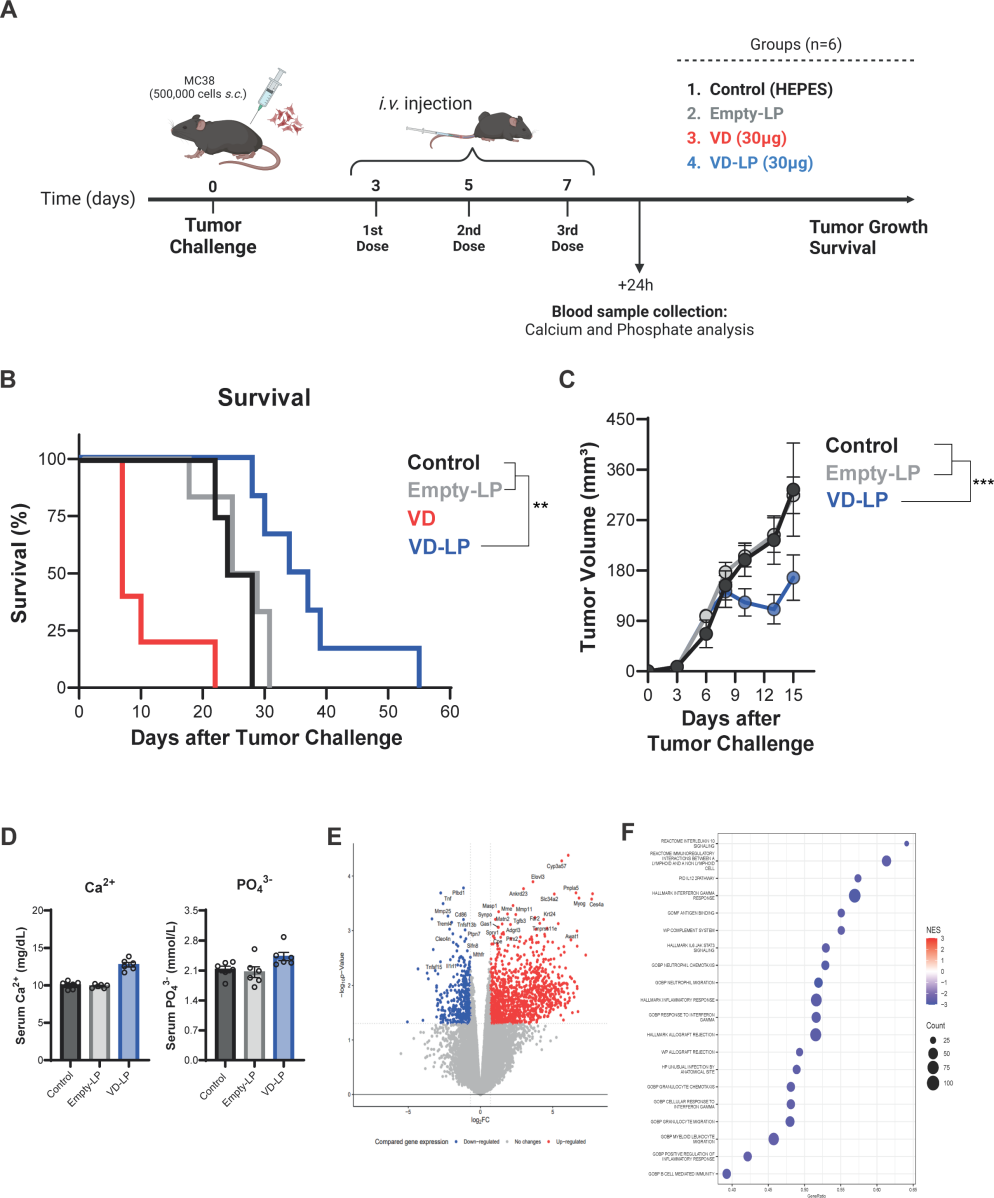
*In Vivo* Antitumor Efficacy and Toxicity of VD-LP in a Mouse Model **(A)** Graphical representation of the *in vivo* assay. C57BL/6J mice were subcutaneously inoculated with 5 × 10^5^ MC38 cells and treated with VD-LP, free VD, Empty-LP, or HEPES buffer (control). **(B)** Kaplan-Meier survival curve for mice treated with VD-LP, free VD, Empty-LP, or HEPES. VD-LP treatment resulted in improved survival rates compared with those of the other groups. Log-rank (Mantel-Cox) test. **(C)** Tumor volume over time in mice treated with VD-LP, free VD, Empty-LP, or HEPES. VD-LP significantly reduced tumor growth compared with that in the control groups. One-way ANOVA with Sidak’s multiple comparisons test was used. *p ≤ 0.05, **p ≤ 0.01, ***p ≤ 0.001. **(D)** Measurement of toxicity: serum calcium (Ca^2+^) and phosphate (PO_4_^3-^) levels. **(E)** Volcano plot of differentially expressed genes in tumor tissues from VD-LP-treated versus control mice, highlighting upregulated and downregulated genes. **(F)** GSEA plot showing significant pathways that were activated or inhibited in tumor tissues following VD-LP treatment.

To evaluate the antitumor efficacy of VD-LP *in vivo*, we employed a mouse model in which MC38 colon carcinoma cells were subcutaneously implanted. The mice were treated with VD-LP, VD, Empty-LP, or HEPES buffer as a control (**Figure 4A**). The administration of VD induced acute toxicity, with only one animal surviving long enough to assess tumor growth (**Figure 4B**). In the remaining experimental groups, no early signs of acute toxicity were observed, allowing for the evaluation of tumor progression. Compared with control mice (median survival time: 26 days) and Empty-LP-treated mice (median survival time: 26 days), which exhibited similar survival rates, those treated with VD-LP showed significantly reduced tumor growth. Moreover, the survival of the VD-LP-treated group was significantly prolonged, with a median survival time of 35.5 days (p < 0.01 when compared to the control group) (**Figures 4B, C**).

To evaluate the potential toxicity of VD-LP, we analyzed the serum calcium and phosphate levels. Interestingly, VD-LP treatment did not cause significant changes in these parameters, suggesting a favorable toxicity profile (**Figure 4D**). These results underscore the enhanced safety and therapeutic efficacy of the liposomal formulation.

Gene expression analysis was conducted on tumor tissues from VD-LP-treated and control mice to investigate the molecular mechanisms underlying the observed antitumor effects. RNA-seq data revealed significant upregulation and downregulation of genes involved in cell proliferation, apoptosis, and immune response pathways following VD-LP treatment. Volcano plot analysis revealed clear differences in gene expression profiles between the VD-LP-treated group and the control group (**Figure 4E**). Furthermore, gene set enrichment analysis (GSEA) revealed significant downregulation of multiple pathways related to the immune response and inflammation (**Figure 4F**). These findings suggest that VD-LP exerts its antitumor effects by modulating key inflammatory pathways within the tumor microenvironment.

## 4 Discussion

This study highlights the potential of liposomal encapsulation to enhance the therapeutic efficacy and safety profile of vitamin D_3_ (cholecalciferol) for cancer treatment. Our findings demonstrate that VD-LP exhibits superior stability, enhanced biological activity, and significant anticancer effects both *in vitro* and *in vivo*, while minimizing the toxicity typically associated with free vitamin D_3_. These results extend the current knowledge on the advantages of liposomal drug delivery systems, particularly in improving the pharmacokinetics and therapeutic outcomes of hydrophobic drugs such as vitamin D_3_ (18, 24, 25).

The physicochemical stability of VD-LP, as evidenced by the consistent particle size, polydispersity index (PDI), and encapsulation efficiency (EE) over 90 days at 4°C, suggests that this formulation is highly stable under storage conditions. Our data indicate that VD-LP maintained a mean particle size of ~83 nm with a narrow size distribution (PDI < 0.05) and high EE (~95%). These findings align with previous studies indicating that liposomal encapsulation can improve the absorption of hydrophobic drugs such as vitamin D_3_, which are otherwise prone to degradation in oily formulations (20). The uniform size and spherical morphology of VD-LP, as confirmed by transmission electron microscopy (TEM), further support its potential for controlled drug delivery, with small, well-defined particles facilitating better biodistribution and tumor-targeting properties (8). In terms of biological activity, gene expression analysis of THP-1 cells revealed that VD-LP significantly modulated the expression of genes involved in the immune response and metabolism. Notably, compared with free vitamin D_3_, VD-LP treatment led to the upregulation of the cathelicidin antimicrobial peptide (hCAMP) and vitamin D receptor (hVDR) genes, suggesting enhanced immune modulation. This aligns with earlier findings that vitamin D_3_ induces antimicrobial peptide expression, although the liposomal formulation appeared to further enhance this effect, likely due to improved cellular uptake and sustained release. The limited activity observed in the free vitamin D_3_ treatment group may be explained by reduced intracellular delivery in the presence of serum. Our previous research has demonstrated that scavenger receptor class B type I is required for 25-hydroxycholecalciferol cellular uptake and signaling. In serum-rich conditions, the lipoproteins may restrict its cellular entry and subsequent activity (26). The liposomal formulation appears to bypass these limitations, facilitating more efficient intracellular delivery and enhancing stability, which likely accounts for the observed differences in gene expression profiles between VD-LP and free vitamin D_3_ treatments.

The modulation of immune-related genes and pathways, such as hCAMP and VDR, by VD-LP treatment may have implications for its potential role in antitumor responses. These genes are known to influence the immune microenvironment, particularly by enhancing antimicrobial peptide expression and promoting the activation of the vitamin D receptor, which plays a role in immune regulation. Enhanced expression of these genes could contribute to a more robust immune activation, potentially facilitating tumor immune surveillance and control (27, 28).

The observed upregulation of metabolic genes, such as ABCD2 and FBP1, following VD-LP treatment, suggests that the liposomal formulation may influence key pathways in cancer metabolism. ABCD2 is part of the peroxisomal transporter family and has been implicated in the regulation of lipid metabolism, which is critical for energy homeostasis and cellular proliferation in cancer. Enhanced ABCD2 expression may reflect a shift in the metabolic state of tumor cells toward pathways less favorable for tumor progression, potentially through altered fatty acid oxidation or lipid biosynthesis (29, 30). Similarly, FBP1, a gluconeogenesis-related enzyme, has been associated with tumor suppression in various cancers. Its upregulation may disrupt the glycolytic phenotype typically exhibited by cancer cells, known as the Warburg effect, thereby impairing their metabolic adaptability and proliferation (31–33). These findings point to a broader antitumor potential of VD-LP, not only through immune modulation but also by altering cancer cell metabolism (34). Future studies could investigate whether these changes in metabolic gene expression directly contribute to tumor growth inhibition or interact synergistically with immune pathways to enhance therapeutic efficacy. This line of research could provide novel insights into the metabolic vulnerabilities of tumors and inform the development of combination therapies targeting both immune and metabolic axes.

Compared with those of untreated controls, the antiproliferative effects of VD-LP on multiple cancer cell lines—HT29, BT474, and TRAMP-C1—were particularly striking, with significant reductions in cell proliferation. These findings build on the work of Krishnan et al., who demonstrated the antiproliferative effects of vitamin D_3_ on various cancer cell lines through cell cycle arrest and apoptosis (35). However, VD-LP was more effective than free vitamin D_3_, suggesting that liposomal encapsulation enhances drug efficacy, likely by improving cellular delivery and therefore increasing the intracellular availability of vitamin D_3_. This finding is consistent with other studies demonstrating the enhanced efficacy of liposomal formulations, such as liposomal paclitaxel and liposomal doxorubicin, compared with their free forms (36).

In an *in vivo* MC38 colon carcinoma mouse model, compared with free vitamin D_3_ and control treatments, VD-LP treatment significantly inhibited tumor growth and improved survival rates. These findings underscore the potential of VD-LP as a more potent antitumor agent, with enhanced bioavailability and efficacy due to the liposomal delivery system. The reduced toxicity of VD-LP, as evidenced by the absence of hypercalcemia and weight loss, further supports its therapeutic advantage over free vitamin D_3_, which is often associated with toxicity at high doses. This favorable toxicity profile suggests that VD-LP allows for increased therapeutic dosing without the adverse effects typically linked to high-dose vitamin D_3_ administration (37).

Gene expression analysis of tumor tissues provided additional insights into the molecular mechanisms underlying the antitumor effects of VD-LP. Significant modulation of genes involved in cell proliferation, apoptosis, and immune response pathways was observed following VD-LP treatment. Notably, GSEA revealed downregulation of immune and inflammatory pathways, suggesting that VD-LP may also exert its antitumor effects by modulating the tumor microenvironment. These findings support previous studies demonstrating the immunomodulatory potential of vitamin D_3_ and its role in enhancing the immune response against tumors (38). The ability of VD-LP to influence both tumor-intrinsic and immune-related pathways reinforces its potential as a powerful anticancer agent.

To advance the translational potential of the VD-LP formulation, future efforts should focus on defining a clinically viable target product profile. Intravenous administration appears most suitable for systemic delivery, ensuring efficient distribution and bioavailability while minimizing first-pass metabolism. Alternatively, subcutaneous administration could be explored for its convenience in outpatient settings or for use in extended-release formulations. Regarding formulation, a lyophilized product format would offer advantages in terms of long-term stability and ease of storage and transportation. This format could be reconstituted into an injectable solution prior to administration. The final dosage and volume of administration will require optimization to ensure safety and efficacy, particularly in preventing hypercalcemia while maintaining therapeutic benefits. The scalability of the VD-LP formulation is promising, as established liposomal manufacturing technologies, such as high-pressure homogenization or ethanol injection methods, are already compliant with pharmaceutical production standards. These features support the potential for large-scale production and clinical application. Targeting VD-LP as an adjunctive therapy in cancer or other diseases leveraging its immunomodulatory and antitumor properties could position it as a valuable addition to existing therapeutic regimens. Future studies should aim to evaluate its pharmacokinetics, pharmacodynamics, and compatibility with current treatment modalities to refine its clinical potential and ensure its successful translation to patient care. VD-LP offers distinct advantages over other strategies for mitigating vitamin D_3_ toxicity. Unlike analogues, which require chemical modification and may lack the full biological activity of native vitamin D3, liposomal encapsulation preserves its natural structure while enhancing bioavailability and reducing systemic toxicity (10). Compared to combination therapies, which involve additional agents and require precise dose calibration, VD-LP provides a simpler and more direct approach, ensuring sustained release and targeted delivery (17). These features make liposomal encapsulation a scalable and versatile solution, positioning it as a promising strategy for reducing toxicity while maintaining the therapeutic potential of vitamin D_3_, particularly in oncology.

The carrier material DSPE-PEG2000, widely used for its biocompatibility and low immunogenicity, is not entirely free from immunogenic potential. Reports of anti-PEG antibody development and hypersensitivity reactions highlight the need for safety considerations. While our study focused on acute toxicity and efficacy, no immediate adverse effects related to DSPE-PEG2000 were observed. However, long-term immunogenicity assessments are crucial, particularly for repeated dosing. Future strategies, such as optimizing PEG density or exploring alternative materials, could further enhance the formulation’s safety and clinical viability.

While our study demonstrates the potential of VD-LP in reducing toxicity and enhancing antitumor efficacy, several limitations should be acknowledged. First, the long-term safety and immunogenicity of the liposomal formulation, particularly with repeated dosing, remain to be thoroughly evaluated. Second, although VD-LP was compared to free vitamin D3 in this study, direct comparisons with other advanced drug delivery systems were not included and could provide additional context for its relative advantages. Third, the mechanistic pathways underlying the improved efficacy and reduced toxicity of VD-LP require further exploration to fully understand its therapeutic potential. Finally, the efficacy of VD-LP as a standalone treatment is limited. Vitamin D_3_, including its liposomal formulation, may be best suited for use in combination therapies rather than as a monotherapy in cancer treatment. Integration with other modalities, such as chemotherapy, immunotherapy, or targeted therapies, could potentially enhance its antitumor effects by leveraging complementary mechanisms of action. For instance, combining VD-LP with immune checkpoint inhibitors might amplify immune activation, while pairing it with cytotoxic agents could exploit its modulatory effects on tumor metabolism. Addressing these limitations in future studies will strengthen the evidence base and facilitate the translation of VD-LP into clinical applications.

In conclusion, this study demonstrated that liposomal encapsulation of vitamin D_3_ enhances its stability, bioavailability, and therapeutic efficacy while reducing its associated toxicity. VD-LP exhibits potent anticancer activity across multiple cancer cell lines and in an *in vivo* tumor model, making it a promising candidate for future clinical applications. The ability of VD-LP to modulate key molecular pathways involved in tumor growth and the immune response further supports its potential as a novel therapeutic approach for cancer treatment. Future research should focus on optimizing the formulation and exploring its effects in clinical settings to fully realize its therapeutic potential.

## Data Availability

The datasets presented in this study can be found in online repositories. The names of the repository/repositories and accession number(s) can be found in the article/supplementary material.
